# Nurse-led enhanced recovery program for women undergoing elective cesarean section: a quasi-experimental study

**DOI:** 10.1186/s12912-026-04528-9

**Published:** 2026-04-11

**Authors:** Reda Mohamed-Nabil Aboushady, Mastoura Khames Farag Gaballah, Basma Mohamed Osman, Walaa M. Abdelrahman, Mona Mohamed Elsayed, Lamia Mohamed-Nabil Ismail, Safa Gaber Salem

**Affiliations:** 1 Maternal and Newborn Health Nursing, Faculty of Nursing, Cairo University, Cairo, Egypt; 2 Medical Surgical Nursing, Al-Moosa College of Health Science, Al-Ahsa, KSA Saudi Arabia; 3 Community Health Nursing, Faculty of Nursing, Cairo University, Cairo, Egypt; 4 Lecturer of Maternal and Newborn Health Nursing, Faculty of Nursing, Cairo University, Cairo, Egypt; 5 Medical Surgical Nursing, Faculty of Nursing, British University in Egypt, El Sherouk City, Egypt; 6 Faculty of Nursing, Nursing Education, Cairo University, Cairo, Egypt; 7 Maternity and Pediatric Health Nursing Department, College of Nursing, Princess Nourah Bint Abdulrahman University, Riyadh, Saudi Arabia

**Keywords:** Nurse-led enhanced recovery program, Cesarean section, Functional independence, Patient satisfaction, Hospital stay

## Abstract

**Background:**

Cesarean sections (CS), although commonly performed and generally considered safe, may be associated with prolonged hospitalization, increased postoperative pain, and a higher risk of complications. Accordingly, this study aimed to evaluate the effectiveness of a nurse-led Enhanced Recovery Program (ERP) on selected outcomes among women undergoing elective CS.

**Methods:**

A quasi-experimental study was conducted across inpatient and outpatient settings at Cairo University Obstetrics and Gynecology Hospital, including 90 women allocated into a study group (*n* = 45) and a control group (*n* = 45). Data was collected using structured interviews, numerical pain rating scale, postoperative recovery criteria, functional outcome assessment, and satisfaction questionnaire.

**Results:**

Both groups had comparable demographic characteristics. The ERP group demonstrated significantly better outcomes including reduced pain scores (2.71 vs. 4.26 at discharge, *p* < 0.001), faster ambulation (4.37 vs. 8.11 h), shorter hospital stays (9.22 vs. 11.88 h), and zero wound infections compared to 13.3% in controls. The intervention group showed higher satisfaction rates (33.3% vs. 8.9% completely satisfied) and greater functional outcomes in physical activities, self-care, and baby care 48 h post-discharge (*p* < 0.0001).

**Conclusion:**

The nurse-led ERP effectively improved post-cesarean outcomes, including pain control, recovery time, functional independence, and maternal satisfaction with self and infant care. The findings support integrating nurse-led ERP into cesarean section protocols across maternity hospitals to enhance maternal and neonatal outcomes.

**Clinical trial number:**

Not applicable.

**Supplementary Information:**

The online version contains supplementary material available at 10.1186/s12912-026-04528-9.

## Background

Elective cesarean section (CS) is one of the most commonly performed surgical procedures worldwide, with more than 23 million operations conducted annually [World Health Organization (WHO)] [1]. Globally, approximately one in five births (18.6%) occurs via CS, with particularly high and increasing rates in low and middle-income countries [2, 3]. The global cesarean section rate is expected to reach 29% by 2030, emphasizing the importance of interventions that improve recovery outcomes and resource efficiency [4, 5].

Compared with vaginal birth, cesarean delivery is associated with longer hospital stays and an increased risk of postoperative complications, including excessive bleeding, surgical site and urinary tract infections, delayed initiation of breastfeeding and skin-to-skin contact, and adverse outcomes in subsequent pregnancies [6]. These challenges highlight the importance of implementing structured, evidence-based perioperative care pathways tailored specifically to cesarean section.

Enhanced Recovery Program (ERP) protocols have recently been adapted for cesarean delivery as a multidisciplinary, evidence-based approach aimed at improving maternal recovery while maintaining safety. Cesarean-specific ERP programs standardize nursing and medical care across the preoperative, intraoperative, postoperative, and post-discharge phases, with a focus on early mobilization, effective pain control, early feeding, and maternal–infant bonding. Evidence indicates that ERP in cesarean sections accelerates recovery, improves patient experience, and reduces hospital stay, complications, readmissions, and healthcare costs [7–9].

However, the successful implementation of ERP for cesarean delivery remains challenging, as it requires coordinated multidisciplinary collaboration and a proactive approach to perioperative care. Nurses play a pivotal role in the success of cesarean ERP pathways through patient education, continuous assessment, pain management, and support of early maternal self-care and infant care practices, working alongside anesthesiologists, obstetricians, and hospital administrators [10].

Nurses play a pivotal role in the successful implementation of Enhanced Recovery Programs (ERP) by delivering comprehensive care across the perioperative continuum. Their responsibilities encompass patient counselling, preoperative education, and postoperative management, with a direct influence on patient outcomes through interventions such as effective pain control and early mobilization [11–13]. Through care coordination and continuity, nurses are central to achieving optimal ERP implementation. Moreover, higher adherence to ERP protocols has been associated with reduced nursing workload and improved patient outcomes [14]. Accordingly, this study aimed to evaluate the effectiveness of a Nurse-led Enhanced Recovery Program (ERP) on selected outcomes among women undergoing elective cesarean section.

## Significance of the study

CS is a commonly performed surgical procedure worldwide and is associated with postoperative challenges, including pain, prolonged hospital stays, breastfeeding difficulties, and delayed maternal-infant bonding [15, 16]. ERP programs have been shown to improve postoperative outcomes by reducing complications, accelerating recovery, and enhancing patient experience [8]. Despite this, research on nurse-led ERP interventions for women undergoing CS remains limited [17, 18].

Most existing studies focus on short-term clinical outcomes, such as wound healing, pain control, and complication prevention, with little attention to holistic recovery that integrates physical, psychological, nutritional, and educational dimensions. Furthermore, interventions are often medically oriented rather than nursing-led and may lack culturally appropriate strategies to address women’s unique needs. Long-term outcomes beyond the immediate postoperative period are also underexplored, particularly in developing countries.

This study addresses these gaps by developing and evaluating a structured, nursing-led recovery program for women undergoing CS. By focusing on holistic recovery, including physical, psychological, nutritional, and educational aspects, the program aims to improve maternal outcomes, enhance quality of life, and support early maternal-infant bonding. The findings will provide evidence-based guidelines for nursing practice, inform culturally sensitive interventions in developing countries, and contribute to optimizing post-cesarean care standards while supporting policymakers and healthcare providers in designing effective maternal care programs.

### The aim of the study

This study aims to evaluate the effectiveness of a Nurse-led Enhanced Recovery Program (ERP) on selected outcomes among women undergoing elective cesarean section.

## Operational definitions

### Selected outcomes

 It includes: (a) Pain intensity; (b) Maternal postoperative recovery criteria in terms of early ambulation, restore bowel motility, first passage of flatus, urinary catheter removal, and length of hospital stay; and postoperative complications and decrease hospital readmission (c) Maternal satisfaction; (d) Maternal functional outcomes in terms of physical activity, self-care activity and baby care.

### Research hypotheses

To fulfill the aim of this study, the following research hypothesis was formulated: Women who receive nurse-led ERP will have improved outcomes compared with those who receive routine care.

### Sub- hypotheses

- H1: Women enrolled in the nurse-led ERP will report significantly lower postoperative pain scores than those in the routine care group.
- H2: The intervention group will exhibit enhanced adherence to and improvement in standardized postoperative recovery criteria compared to the control group.”
- H3: Maternal satisfaction levels regarding perioperative care will be significantly higher among women receiving the nurse-led ERP.
- H4: The nurse-led ERP group will demonstrate superior functional performance in physical activity, self-care, and neonatal care at 48 h post-discharge.
- H5: Implementation of the nurse-led ERP will be associated with a reduction in postoperative complications and lower rates of hospital readmission compared to conventional care.

## Methods

### Research design

A quasi-experimental study was utilized to evaluate the effect of nurse-led ERP on outcomes among women undergoing elective cesarean section.

### Setting

This quasi-experimental study was conducted across inpatient and outpatient settings at Cairo University Obstetrics and Gynaecology Hospital, a 297-bed tertiary care teaching hospital in Cairo, Egypt. Inpatient services included six specialized obstetrics departments with 175 beds, two caesarean theatres, and post-operative recovery units. Outpatient components utilized the antenatal clinic with prenatal care facilities and ultrasound services. Both settings ensured care continuity from pre-operative visits through 48-hour post-discharge follow-up. The hospital’s 24/7 services provided optimal conditions for implementing the nurse-led Enhanced Recovery Program across the complete perioperative continuum.

### Sample

A total of 90 women scheduled for elective cesarean section were recruited using purposive sampling and allocated to either the study group (*n* = 45) or the control group (*n* = 45). The control group received routine hospital care, while the study group participated in a nurse-led Enhanced Recovery Program (ERP). The sample size was calculated using G*Power version 3.1, with a power of 0.95, an alpha level of 0.05 (one-sided), and an effect size of 0.80, ensuring a 95% probability of detecting true ERP effects.

Inclusion criteria comprised women undergoing elective cesarean section at term, primiparous with a single viable pregnancy, aged 18–45 years, with a BMI below 30, and no history of medical conditions or chronic pain within the past year. Exclusion criteria included combined surgical procedures, multiple gestations, bleeding disorders, abnormal placental implantation, and cases requiring general anesthesia.

### Tools of data collection

Data was collected using five tools (Supplementary File 1):

### Tool 1: Maternal structured interviewing questionnaire

This tool was developed by the researchers and consists of two parts:

1. **Personal characteristics**: age, educational level, residence, and occupation.
2. **Maternal characteristics**: gestational age, hemoglobin level, BMI, vital signs before and after CS, duration of surgery (minutes), and newborn weight (kg).

### Tool 2: Numerical pain rating scale (NPRS)

The NPRS was adopted from [19] to assess pain intensity. It is a segmented numeric version of the visual analog scale (VAS), where respondents select a whole number from 0 (no pain) to 10 (worst imaginable pain). Pain severity is interpreted as follows: No pain (0), Mild (1–3), Moderate (4–6), Severe (7–10). The NPRS demonstrates high test-retest reliability (*r* = 0.96) [19] and was used three times in this study: 2 h and 4 h postoperatively, and before discharge. Internal consistency in this study was high (Cronbach’s α = 0.89).

### Tool 3: Maternal postoperative recovery criteria

Adapted from [20] and modified by the researchers, this tool evaluates perioperative nursing interventions across three stages: preoperative, intraoperative, and postoperative. It also includes nursing education regarding early ambulation, bowel motility, first passage of flatus, urinary catheter removal, length of hospital stay, and postoperative follow-up (complications and readmission).

### Tool 4: Maternal functional outcomes assessment tool

Developed by the researcher [21], this tool evaluates women’s functional status during the first 48 h postpartum following elective CS (Supplementary File 1). It provides a comprehensive assessment of early postpartum functional recovery, focusing on activities critical to immediate post-surgical rehabilitation and independence.

### Tool Structure and content

The 12-item assessment is organized into three functional domains:

1. **Physical Activities (6 items)**: Evaluates mobility functions from turning in bed to walking and sitting.
2. **Self-Care Activities (4 items)**: Assesses personal care independence, including hygiene, eating, and rest.
3. **Baby Care (2 items)**: Measures ability to breastfeed and provide basic baby care.

These domains capture essential functional areas critical for post-cesarean recovery and maternal role fulfillment.

### Development rationale

The tool was developed to address the need for a targeted assessment instrument that captures the specific functional challenges faced by women during recovery from cesarean delivery. While existing instruments, such as the Inventory of Functional Status After Childbirth (IFSAC) [21, 22], provide general assessments of postpartum functional status, this tool was specifically designed to evaluate the immediate post-cesarean period. It integrates evidence-based concepts from established postpartum functional assessment literature while focusing on the unique requirements of surgical recovery.

### Instrument development and validation

The development process adhered to established psychometric principles. Content validity was confirmed through review by three clinical experts in obstetric nursing and maternal health, who assessed each item for relevance, clarity, and comprehensiveness. The experts agreed that all items were appropriate for evaluating functional status in the early post-cesarean period and that the three-domain structure adequately reflected the key areas of functional recovery.

### Language and cultural considerations

The tool was originally developed in Arabic to ensure cultural relevance and comprehension among the study population. A professional translation service was used to produce the English version provided in Supplementary File 1, with back-translation performed to verify accuracy and equivalence between versions.

### Scoring system

Scoring reflects the level of independence in performing each activity:

1. Inability to perform the activity.
2. Partial dependence.
3. Full independence.

The total score ranges from 12 to 36, capturing the full spectrum of functional abilities during early postpartum recovery. Scores are interpreted as follows: 12– ≥12 indicates inability to perform activities, 13–24 indicates partial dependence, and 25–36 indicates full independence.

### Tool 5: Maternal satisfaction with the ERP

To evaluate maternal satisfaction with the nurse-led Enhanced Recovery Program (ERP) following elective cesarean section, a 10-point scale was adopted from [23]. This scale captures participants’ satisfaction with various aspects of their care.

### Scoring system

- The scale is anchored with descriptive labels at each end: 0 = “Not at all satisfied” and 10 = “Completely satisfied.”
- Intermediate scores are interpreted as follows: 0–4 = “Not satisfied,” 5 = “Neutral,” 6–9 = “Somewhat satisfied,” and 10 = “Completely satisfied.”

#### Validity and reliability

For validity, tools were reviewed by a panel of five experts in Medical-Surgical, Community Health, and Maternity Health Nursing. The panel assessed face and content validity, clarity, and content appropriateness. The tools were then revised based on the panel’s feedback. The consistency of the tools was evaluated using a test-retest method with 10 women. Internal consistency was then measured using Cronbach’s alpha. Acceptable reliability was defined as a coefficient greater than 0.7. Cronbach’s alpha coefficients for the maternal structured interview questionnaire, postoperative recovery criteria, functional outcomes, and satisfaction were 0.78, 0.82, and 0.88, respectively, indicating acceptable reliability.

### Procedure

This study was conducted over nine months and involved 90 women undergoing elective cesarean sections. A nurse-led Enhanced Recovery Program (ERP) was developed in Arabic, informed by a comprehensive review of Arabic and English literature, to optimize maternal outcomes. Data collection and ERP implementation were structured into three sequential phases, complemented by additional program components to ensure comprehensive care and strong data collection.

#### Phase I: Initial assessment and baseline data collection

Following ethical approval and administrative arrangements with the hospital, participants were recruited into either the control group, receiving standard care, or the study group, receiving the ERP. To minimize potential contamination, the control group was recruited and assessed first at the antenatal clinic.

### Control group

- Baseline data, including demographic and maternal characteristics (e.g., age, parity, gestational age, and relevant medical history), were collected before surgery.
- Postoperatively, pain intensity and adherence to routine recovery criteria were monitored every two hours until discharge, documenting the standard care provided by nursing staff.

### Study group

- The study sample received preoperative counseling detailing the ERP to ensure informed consent and understanding.
- Baseline assessments were conducted within two hours post-Cesarean section, including:
  - Vital signs.
  - Pain intensity (assessed using the Visual Analog Scale – VAS).
  - Time to first flatus.
  - Breastfeeding initiation.
- A second assessment occurred at four hours post-Cesarean section, evaluating:
  - Pain intensity (VAS).
  - Early ambulation.
  - Bowel motility.
  - Time to first flatus.
  - Urinary catheter removal.

#### Phase II: Implementation of the enhanced recovery program (ERP)

**Fig. 1:**
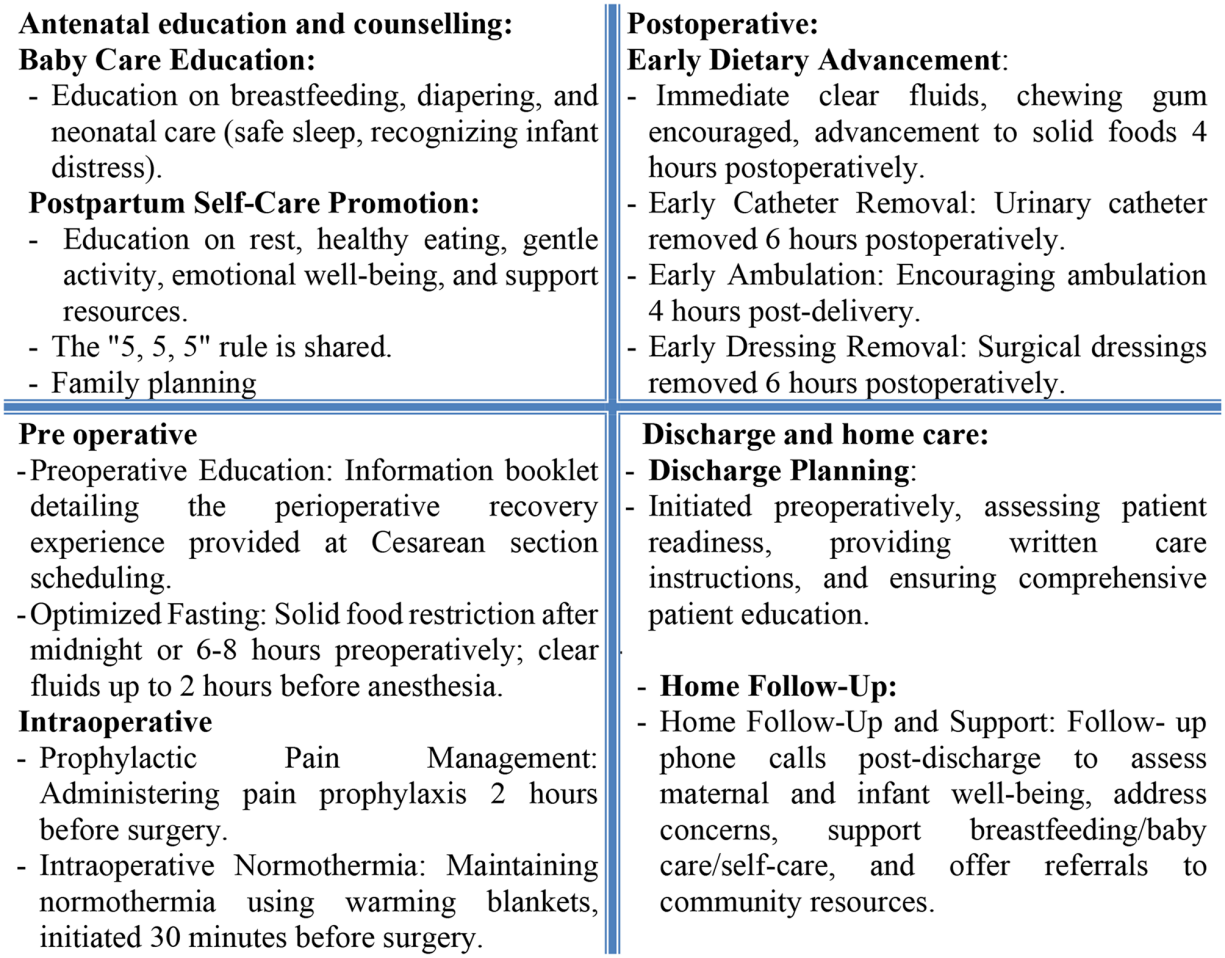
Components of nurse-led enhanced recovery program (**ERP)** for women undergoing Elective Cesarean Section

- Control Group: The researcher’s role was observational, documenting standard perioperative care provided by nursing staff.
- Study Group: The researcher implemented the key components of the nurse-led ERP, spanning from the antenatal phase through discharge and home follow-up (Fig. 1). Core ERP components included:
  - Preoperative education and counseling.
  - Early mobilization and ambulation.
  - Pain management strategies.
  - Nutrition and hydration optimization.
  - Bowel and bladder care.
  - Breastfeeding support.
  - Discharge planning and home follow-up guidance.

#### Phase III: Post-discharge evaluation and follow-up

Postoperative outcomes, including pain intensity (VAS), maternal satisfaction, and length of hospital stay, were recorded on the day of discharge for both groups. Following discharge, participants were contacted via phone and WhatsApp to assess functional recovery, monitor postoperative complications, and track hospital readmissions. Each follow-up call lasted approximately 15–20 min per participant. During these interactions, participants could ask questions, and the researcher documented any concerns or needs, providing referrals to outpatient clinics as appropriate.

### Additional components of the enhanced recovery program (ERP)

In addition to the structured phases, the ERP incorporated the following elements to ensure comprehensive care and rigorous data collection:

- Pain Assessment: Pain intensity was measured using the VAS at three time points: baseline (within two hours post-Cesarean), four hours post-Cesarean, and at discharge.
- Continuous Monitoring: Maternal and infant well-being were continuously assessed throughout the program to ensure safety and timely intervention.
- Documentation: All interventions, assessments, and outcomes were systematically recorded to maintain accurate and complete data.
- Multidisciplinary Collaboration: The ERP was implemented in coordination with a multidisciplinary team, including obstetricians, anesthetists, pediatric nurses, and physiotherapists, to optimize maternal and neonatal outcomes.

### Ethical considerations

The study received approval from Cairo University’s Faculty of Nursing Ethical Research Committee IRB 2,019,041,701. Participants received detailed information sheets and provided written informed consent. They were assured of their right to withdraw without consequences, and all data was coded for anonymity and stored securely. Personal information was protected and used solely for this research, with any additional use requiring participant permission. These measures ensured ethical compliance throughout the study.

### Statistical data analysis

Data was coded, entered, and analyzed using the Statistical Package for the Social Sciences (SPSS), version 20. The results were presented in tabular form. Descriptive and inferential statistical analyses were conducted, including frequencies, percentages, means, and standard deviations. The independent *t*-test was used to compare mean differences between groups, while the chi-square test was applied to examine associations between categorical variables. Statistical significance was set at a *p*-value ≤ 0.05.

## Results

**Table 1:** Frequency and percentage distribution of personal characteristics among the study and control groups (*n* = 90)

Characteristics	Control		Study		T / χ² (*P*-value)
	No.	%	No.	%	
**Age (years)**					
18–24	21	46.7	14	31.1	2.33 (0.31)
25–29	10	22.2	12	26.7	
≥ 30	14	31.1	19	42.2	
(Mean ± SD) yrs.	26.24 ± 5.73		27.88 ± 5.23		1.42 (0.15)
**Residence**					
Urban	26	57.8	23	51.1	0.40 (0.52)
Rural	19	42.2	22	48.9	
**Education**					
Read and write	13	28.9	20	44.4	6.14 (0.18)
Primary education	6	13.3	2	4.4	
Preparatory education	7	15.6	10	22.2	
Secondary education	15	33.3	12	26.7	
University education	4	8.9	1	2.2	
**Occupational level**					
Housewife	36	80	37	82.2	0.07 (0.78)
Working	9	20	8	17.8	

Table 1 shows that the mean age was comparable between groups (26.24 ± 5.73) years in the control group versus (27.88 ± 5.23) years in the study group. More than half of the study sample in both groups resided in urban areas (57.8% in the control group and 51.1% in the study group). Women with secondary education constituted the largest proportion in both groups (33.3% in the control group and 26.7% in the study group). Illiteracy or basic literacy (ability to read and write) was more common in the study group (44.4%) than in the control group (28.9%). Notably, the vast majority of the study and control groups were housewives (80% and 82.2%), respectively. Overall, no statistically significant differences were observed between the study and control groups in personal characteristics.

**Table 2:** Frequency and percentage distribution of maternal characteristics among the study and control groups (*n* = 90)

Variables			Control			Study			T / χ² (*P*-value)
			No.	%		No.	%		
**Gestational (in week)**			38.68± 1.37			38.31 ± 0.97			-1.50 (0.13)
**Hemoglobin level**			9.22 ± 1.50			9.24 ± 1.55			-0.06 (0.94)
**BMI**:									
Normal			12	26.7		11	24.4		0.09 (0.95)
Overweight			23	51.1		23	51.1		
Obese			10	22.2		11	24.4		
Mean ± SD			27.48 ± 5.20			27.75 ± 5.02			0.24 (0.80)
**Vital signs before and after CS (Mean ± SD)**:									
Temperature	Before	37.00 ± 0.30			36.99 ± 0.21			0.03 (0.90)	
	After	37.00 ± 0.30			36.99 ± 0.21			1.50 (0.13)	
Pulse	Before	90.97 ±11.20			100.68 ± 12.24			0.61 (0.54)	
	After	86.57 ± 10.48			83.15 ± 11.07			− 0.12 (0.90)	
Systolic BP	Before	99.75 ± 16.56			103.77 ± 9.11			1.42 (0.15)	
	After	103.33 ± 10			108.44 ± 11.47			2.25(0.02)*	
Diastolic BP	Before	63.51 ± 6.68			64.66 ± 6.60			0.36 (0.41)	
	After	64.26 ± 5.48			72.84 ± 6.39			6.83 (0.01)*	
Respiration	Before	22.57 ± 2.58			21.44 ± 1.77			− 0.12 (0.90)	
	After	22.57 ± 2.58			21.44 ± 1.77			− 0.12 (0.90)	
**Duration of surgery (in minutes)**		42.02 ± 10.93			40.82 ± 11.42			0.050 (0.61)	
**Baby Weight (Kg.)**		2.57 ± 0.63			3.09 ± 0.59			3.98 (0.001)**	

*Statistically significant p-value ≤ 0.05

Table 2 indicates that there were no statistically significant differences between the control and study groups regarding gestational age, hemoglobin level, BMI categories, mean BMI, duration of surgery, or most pre- and post-cesarean vital signs (*p* > 0.05), indicating baseline clinical comparability. Maternal temperature, pulse rate, respiratory rate, and systolic and diastolic blood pressure before cesarean section were similar in both groups. However, postoperative systolic and diastolic blood pressure were significantly higher in the study group (*p* < 0.05). In addition, neonatal birth weight was significantly greater among the study group compared with the control group (*p* = 0.001).

**Table 3:** Frequency and percentage distribution of pain intensity after cesarean section among the study and control groups (*n* = 90)

Variables	Control		Study		T / χ² (*P*-value)
	No.	%	No.	%	
Pain 2 h. postoperative:					
Mild	0.00	0.00	0.00	0.00	3.08 (0.07)
Moderate	2	4.4	7	15.6	
Severe	43	95.6	38	84.4	
Overall mean pain score	8.86 ± 0.75		8.40 ± 0.93		− 2.59 (0.01)*
**Pain 4 h. postoperative**:					
Mild	12	26.7	29	64.4	13.25 (0.001) **
Moderate	22	48.9	12	26.7	
Severe	11	24.4	4	8.9	
Overall mean pain score	5.35 ± 2.02		3.84 ± 0.85		− 4.16 (0.001) **
**Pain before discharge**:					
Mild	19	42.2	39	86.7	19.39 (0.001) **
Moderate	26	57.8	6	13.3	
Severe	0	0.00	0	0.00	
Overall mean pain score	4.26 ± 1.54		2.71 ± 0.69		− 6.16 (0.001) **

*Statistically significant p-value ≤ 0.05

**Highly statistically significant p-value ≤ 0.01

Table 3 Post-cesarean pain data showed similar severe pain in both groups at 2 h post-operation. Postoperative pain was significantly lower in the study group compared with the control group, particularly at four hours postoperatively and before discharge. Overall pain scores showed a consistent and statistically significant reduction in the study group across the postoperative period.

**Table 4:** Frequency and percentage distribution of postoperative recovery criteria among the study and control groups (*n* = 90)

Variables	Mean duration in hours after CS		T (*P*-value)
	Control	Study	
Early ambulation.	8.11 ± 2.41	4.37 ± 0.61	-10.05 (0.01) *
Restore bowel motility.	4.26 ± 2.64	2.62 ± 1.24	3.77 (0.01) *
First passage of flatus.	8.31 ± 3.85	5.51 ± 2.59	− 4.04 (0.01) *
Urinary catheter removal.	7.62 ± 3.41	4.13 ± 0.50	− 6.77 (0.01) *
Length of hospital stays.	11.88 ± 2.65	9.22 ± 4.02	− 3.70 (0.01) *

*Statistically significant p-value ≤ 0.05

Table 4 Women in the study group experienced significantly earlier postoperative recovery indicators, including the following: early ambulation, return of bowel motility, passage of flatus, and urinary catheter removal, compared with the control group. Moreover, the length of hospital stay was significantly shorter among women in the study group following cesarean section.

**Table 5:** Frequency and percentage distribution of postoperative recovery criteria regarding intraoperative, postoperative complication and hospital readmission among the study and control groups (*n* = 90)

Variables	Control		Study		T / χ² (*P*-value)
	No.	%	No.	%	
Intraoperative complications:					
Yes	6	13.3	3	6.7	1.11 (0.29)
No	39	86.7	42	93.3	
**Reason of intraoperative complications**:					
Vomiting	2	4.4	2	4.4	1.91 (0.38)
Shivering	4	8.9	1	2.2	
**Postoperative complications and hospital readmission**:					
Yes (Reason: Wound infection)	6	13.3	0	0.00	6.42 (0.001) **
No	39	86.7	45	100	

*Highly statistically significant p-value ≤ 0.001

Table 5 No statistically significant difference was observed between the control and study groups regarding the occurrence of intraoperative complications (*p* > 0.05). While, postoperative complications and hospital readmission were significantly lower in the study group as compared with the control group (*p* = 0.001).

**Table 6:** Frequency and percentage distribution of maternal satisfaction among the study and control groups (*n* = 90)

Variables	Control		Study		T / χ² (*P*-value)
	No.	%	No.	%	
Satisfaction level:					10.62 (0.01)*
Not satisfied (1–4)	12	26.7	5	11.1	
Neutral (5)	18	40	12	26.7	
Somewhat satisfied (6–9)	11	24.4	13	28.9	
Completely satisfied (10)	4	8.9	15	33.3	
**Mean ± SD**	**5.35 ± 2.04**		**7.97 ±2.12**		5.96 (0.0001)**

*Statistically significant p-value ≤ 0.05 ** Highly significant p-value ≤ 0.0001

Table 6 Women in the study group reported significantly higher overall satisfaction level regarding ERP as compared with the control group (*χ²* = 10.62, *p* = 0.01). The mean satisfaction score was also significantly greater in the study group (*p* < 0.001).

**Table 7:** Frequency and percentage distribution regarding functional outcomes after 48 h of discharge among the study and control groups (*n* = 90)

Variables	Inability to perform (1) 0 - ≥ 12				Partial dependence (2) 13- ≥ 24				Full independence (3) 25–36				χ² (*P*-value)
	Control		Study		Control		Study		Control		Study		
No	%	No	%	No	%	No	%	No	%	No	%		
**Physical activity**													
Turn in bed	23	51.1	5	11.1	17	37.8	14	31.1	5	11.1	26	57.8	26.08 (0.01) *
Get out of bed	17	37.8	0	0.0	23	51.1	12	26.7	5	11.1	33	73.3	41.08 (0.01) *
Ambulation out of bed	15	33.3	0	0.0	25	55.6	19	42.2	5	11.1	26	57.8	30.04 (0.01) *
Get up from a sitting position	9	20	1	2.2	27	60	11	24.4	9	20	33	73.3	26.85 (0.01) *
Sitting in bed after surgery	12	26.7	1	2.2	29	64.4	6	13.3	4	8.9	38	84.4	51.94 (0.01) *
Return to sleep	14	31.1	5	11.1	23	51.1	14	31.1	8	17.8	26	57.8	15.98 (0.01) *
**Self-care activity**													
Urinate and defecate	12	26.7	0	0.0	26	57.8	13	28.9	7	15.6	32	71.1	32.35 (0.01) *
Change clothes and shower	0	0.0	0	0.0	36	80	11	24.4	9	20	34	75.6	27.83 (0.01) *
Eat	0	0.0	0	0.0	16	35.6	11	24.4	29	64.4	34	75.6	1.32 (0.01) *
Take a nap	5	11.1	0	0.0	22	48.9	6	13.3	18	40	39	86.7	21.88 (0.01) *
**Baby care**													
Breastfeed child	0	0.0	0	0.0	15	33.3	11	24.4	30	66.7	34	75.6	1.39 (0.01) *
Change diaper	0	0.0	0	0.0	5	11.1	2	4.4	40	88.9	43	95.6	0.86 (0.01) *
**Mean ± SD**	5.40 ± 2.52		9.68 ± 2.54		16.84 ± 2.62		19.77 ± 3.46		27.00 ± 2.68		32.51 ± 2.68		
**t-test (p-value)**	8.02 (0.0001)**				4.52 (0.0001)**				9.73 (0.0001)**				

** Highly statistically significant p-value ≤ 0.0001 * Statistically significant differences p-value ≤ 0.05

Table 7 shows that women in the study group demonstrated significantly greater functional independence across all assessed physical, self-care, and baby-care activities compared with the control group (*p* < 0.001). The study group achieved higher mean scores in full independence, while the control group had higher levels of partial dependence or inability.

## Discussion

Enhanced Recovery programs are structured, evidence-based pathways designed to optimize maternal outcomes following cesarean section through multidisciplinary interventions. Unlike routine care, ERP is not simply a collection of postoperative measures; it systematically addresses the physiological, psychological, and functional challenges faced by women after surgery [24–29].

### Maternal and baseline characteristics

Findings of the current study confirmed that the ERP group and control groups were comparable in demographic and clinical characteristics, including age, literacy, BMI, gestational age, and baseline maternal and neonatal parameters. This comparability strengthens the internal validity of the study, allowing observed differences in recovery, pain, and functional outcomes to be confidently attributed to the ERP intervention rather than baseline confounders [30].

### Effect of nurse-led ERP on outcomes

Reductions in postoperative pain, earlier mobilization, and faster restoration of bowel and urinary functions reflect the physiological mechanisms targeted by ERP components. Early ambulation enhances circulation, reduces venous stasis, and stimulates gastrointestinal motility, while early feeding supports metabolic recovery and energy balance [31]. Structured pain management facilitates participation in physical activity, further accelerating functional recovery. These mechanisms explain why ERP participants achieved independence in daily activities and baby care more rapidly than the control group [32].

### Functional recovery and patient-centered outcomes

The findings of the current study indicate that women in the ERP group demonstrated significant improvements in functional activity, self-care, and baby care compared with the control group. These results suggest that postoperative recovery extends beyond biomedical outcomes to encompass functional and psychosocial dimensions. By promoting early mobilization, structured pain management, and timely restoration of bodily functions, ERP facilitates a shift from dependency toward autonomy, enhancing maternal engagement in daily and infant care [33, 34].

The observed improvements can be interpreted as evidence that ERP not only accelerates physical recovery but also strengthens self-efficacy and confidence in performing routine activities. Even modest reductions in pain, when combined with earlier ambulation and bowel function recovery, create cumulative benefits that support functional independence and maternal competence [35]. The rationale for these outcomes lies in the integrated, multidisciplinary nature of ERP, which addresses physiological, psychological, and functional needs simultaneously, allowing women to regain control over their daily activities and caregiving responsibilities more efficiently than with routine postoperative care.

### Postoperative complications and infection control

The baseline infection rate in the routine care group exceeded international averages, likely reflecting hospital-specific factors such as patient demographics, procedural practices, and local infection control standards [36]. ERP components, including timely catheter removal, early mobilization, and structured hygiene protocols, likely mitigated this risk, demonstrating the impact of systematic, evidence-based interventions on clinical outcomes. These findings illustrate that contextual factors interact with intervention efficacy and that ERP benefits may be more pronounced in settings with higher baseline complication rates.

Consistent with prior studies, ERP implementation was associated with reduced length of hospital stay, lower postoperative complications, faster functional recovery, and higher patient satisfaction [28–32, 35–37]. Mechanistically, these improvements reflect the synergistic effect of coordinated perioperative interventions, including early mobilization, structured nutrition, pain control, and patient education, which together accelerate both physical and functional recovery.

## Conclusion

Nurse-led Enhanced Recovery Programs (ERP) constitute a rigorously grounded, evidence-based approach to facilitating maternal recovery following elective cesarean sections. By systematically addressing postoperative pain, promoting early mobilization, and supporting functional independence, these interventions accelerate recovery and enhance patient-reported outcomes. The findings emphasize the critical role of structured, multidisciplinary perioperative care and provide strong justification for the wider adoption of ERP protocols to optimize both clinical and functional outcomes in obstetric practice.

### Implications for nursing education, research and practice

#### Nursing education

- Integrate Enhanced Recovery Program (ERP) principles into undergraduate and postgraduate nursing curricula.
- Emphasize leadership, clinical decision-making, and evidence-based practice in obstetric nursing education.

#### Nursing research

- Encourage further studies on nurse-led ERP across diverse healthcare settings.
- Examine long-term maternal and neonatal outcomes associated with ERP implementation.
- Evaluate the cost-effectiveness and sustainability of nurse-driven recovery models.

#### Nursing practice

- Support the adoption of nurse-led ERP as a standard component of post-cesarean care.
- Empower nurses to take active leadership roles in coordinating multidisciplinary recovery interventions.
- Promote patient-centered care approaches to enhance functional recovery and maternal satisfaction.

### Limitations of the study

1. The quasi-experimental design limited randomization, potentially introducing selection bias and reducing the strength of causal inferences.
2. The short follow-up period precluded assessment of long-term maternal and neonatal outcomes.
3. Measures of pain and satisfaction were self-reported, which may have introduced response bias.
4. Variability in the implementation of the enhanced recovery program by nurses, as well as contextual factors such as maternal comorbidities or family support, may have influenced the study findings.

## Recommendations for future research

Future studies should address these limitations by employing randomized controlled trial designs to strengthen causal inferences. Extending follow-up periods would allow evaluation of the long-term impact of nurse-led ERP on both maternal and neonatal outcomes. Incorporating objective clinical indicators alongside self-reported measures could reduce response bias and provide a more comprehensive assessment of recovery. Furthermore, future research should explore contextual factors, including maternal health and family support, to better understand the conditions under which enhanced recovery programs are most effective and efficient.

## Supplementary Information

Below is the link to the electronic supplementary material.

Supplementary Material 1

## Data Availability

The datasets generated during and/or analyzed during the current study are available from the corresponding author on reasonable request.
