# Supplementary material for: Nurse-led enhanced recovery program for women undergoing elective cesarean section: a quasi-experimental study
Source: BMC Nurs. 2026 Apr 11;25:369. doi: 10.1186/s12912-026-04528-9 (PMC13085533; doi:10.1186/s12912-026-04528-9)
Supplement: Supplementary file 1 — Supplementary Material 1 [file 12912_2026_4528_MOESM1_ESM.pdf]

Nurse-Led Enhanced Recovery Program for Women undergoing Elective Cesarean Section: A quasi-experimental study.

Supplementary File 1

Tools of data collection

The first tool; Maternal Structured Interviewing Questionnaire (developed by researchers)

Demographic and Social Data

- **Mother's Age:** (...............................) years
- **Residence:** Urban □ Rural □
- **Education Level:** Read and write □ Primary □ Preparatory □ Secondary □ University □
- **Occupation:** Housewife □ Working (specify occupation) □

Reproductive History of Current Pregnancy

- **First day of last menstrual period:** (...............................)
- **Expected delivery date:** (...............................)
- **Reasons for planned cesarean delivery:**

  Large baby size □ Abnormal fetal position □ Other (specify) □

Medical data Immediately Before Cesarean Section:

- **Weight: Height:**
- **Body Mass Index BMI:**
- **Vital Signs:**
  - **Blood Pressure:** Normal □ Abnormal □
  - **Pulse:** Normal □ Abnormal □
  - **Temperature:** Normal □ Abnormal □
  - **Respiration:** Normal □ Abnormal □
- **Hemoglobin level at delivery:** (...............................)
- **Platelet count at delivery:** (...............................)
- **Gestational age at delivery:** (...............................)

Medical data During Cesarean Section:

Duration of surgery (in minutes): (...............................)

Type of anesthesia used: General □ Spinal □

Did complications occur in the operating room for the mother:

Bleeding □ Vomiting/regurgitation □ Shivering □ Pain □

Baby's weight: Normal □ Abnormal (specify) □

Nurse-Led Enhanced Recovery Program for Women undergoing Elective Cesarean Section: A quasi-experimental study.

Medical Information Immediately After Cesarean Section and During Hospital Stay (within first 24 hours):

Vital Signs:

- **Blood Pressure:** Normal □ Abnormal □
- **Pulse:** Normal □ Abnormal □
- **Temperature:** Normal □ Abnormal □
- **Respiration:** Normal □ Abnormal □

Pain assessment score:

Pain medications: Diclofenac (dose) □ Pethidine (dose) □ Morphine (dose) □ Paracetamol (dose) □ Other (specify) □

When did first bowel movement begin (in minutes): (……………………….)

When did oral feeding begin (in minutes): (……………………….)

When did getting up from bed begin (in minutes): (……………………….)

When was urinary catheter removed (in minutes): (……………………….)

Duration of hospital stay: One day □ Two days □ Three days □ More (specify) □

Medical data After Cesarean Section (48 hours after delivery):

- **Did any wound complications occur:** Yes (specify) □ No □
- **Did any complications or danger signs occur during postpartum period:** Yes (specify) □ No □
- **Are there any troubles after cesarean section:** Yes (specify) □ No □
- **Did it require returning to the hospital:** Yes (specify) □ No □

Nurse-Led Enhanced Recovery Program for Women undergoing Elective Cesarean Section: A quasi-experimental study.

Second tool: Numerical Pain Rating Scale (NPRS)

Adopted from: Krebs EE, Carey TS, Weinberger M. Accuracy of the pain numeric rating scale as a screening test in primary care. J Gen Intern Med. 2007;22(10):1453-8. doi: 10.1007/s11606-007-0321-2

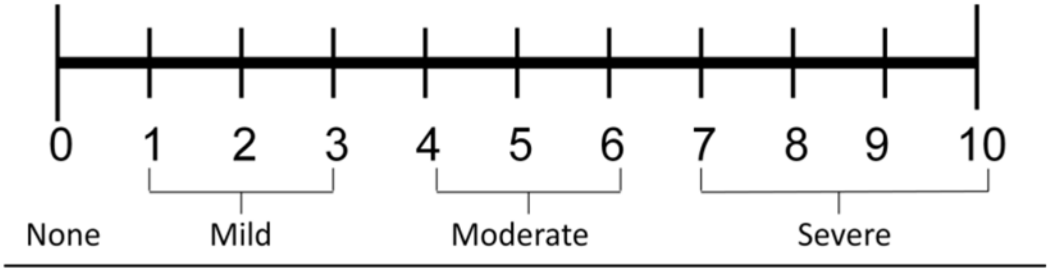

Scoring System

(from 0 no pain at all to 10 worst imaginable pain)

No pain (0);

Mild pain (1-3),

Moderate pain (4-6);

Severe pain (7- 10).

Nurse-Led Enhanced Recovery Program for Women undergoing Elective Cesarean Section: A quasi-experimental study.

Third tool: Maternal Postoperative Recovery Criteria: (developed by researchers)

Indicators	Measurement Time	Strongly disagree ……………………………………………… Strongly agree
**I felt moderate pain**	**After 2 hours**	10 □ 9□ 8□ 7□ 6 □ 5□ 4 □ 3□ 2 □ 1□ 0□
	**After 6 hours**	10 □ 9□ 8□ 7□ 6 □ 5□ 4 □ 3□ 2 □ 1□ 0□
	**After 24 hours**	10 □ 9□ 8□ 7□ 6 □ 5□ 4 □ 3□ 2 □ 1□ 0□
**I felt severe pain**	**After 2 hours**	10 □ 9□ 8□ 7□ 6 □ 5□ 4 □ 3□ 2 □ 1□ 0□
	**After 6 hours**	10 □ 9□ 8□ 7□ 6 □ 5□ 4 □ 3□ 2 □ 1□ 0□
	**After 24 hours**	10 □ 9□ 8□ 7□ 6 □ 5□ 4 □ 3□ 2 □ 1□ 0□
**I experienced nausea or vomiting**	**After 2 hours**	10 □ 9□ 8□ 7□ 6 □ 5□ 4 □ 3□ 2 □ 1□ 0□
	**After 6 hours**	10 □ 9□ 8□ 7□ 6 □ 5□ 4 □ 3□ 2 □ 1□ 0□
	**After 24 hours**	10 □ 9□ 8□ 7□ 6 □ 5□ 4 □ 3□ 2 □ 1□ 0□
**I felt dizzy**	**After 2 hours**	10 □ 9□ 8□ 7□ 6 □ 5□ 4 □ 3□ 2 □ 1□ 0□
	**After 6 hours**	10 □ 9□ 8□ 7□ 6 □ 5□ 4 □ 3□ 2 □ 1□ 0□
	**After 24 hours**	10 □ 9□ 8□ 7□ 6 □ 5□ 4 □ 3□ 2 □ 1□ 0□
**I was shivering**	**After 2 hours**	10 □ 9□ 8□ 7□ 6 □ 5□ 4 □ 3□ 2 □ 1□ 0□
	**After 6 hours**	10 □ 9□ 8□ 7□ 6 □ 5□ 4 □ 3□ 2 □ 1□ 0□
	**After 24 hours**	10 □ 9□ 8□ 7□ 6 □ 5□ 4 □ 3□ 2 □ 1□ 0□
**I was comfortable**	**After 2 hours**	0 □ 1□ 2□ 3□ 4 □ 5□ 6 □ 7□ 8 □ 9□ 10□
	**After 6 hours**	0 □ 1□ 2□ 3□ 4 □ 5□ 6 □ 7□ 8 □ 9□ 10□
	**After 24 hours**	0 □ 1□ 2□ 3□ 4 □ 5□ 6 □ 7□ 8 □ 9□ 10□
**I can move independently**	**After 2 hours**	0 □ 1□ 2□ 3□ 4 □ 5□ 6 □ 7□ 8 □ 9□ 10□
	**After 6 hours**	0 □ 1□ 2□ 3□ 4 □ 5□ 6 □ 7□ 8 □ 9□ 10□
	**After 24 hours**	0 □ 1□ 2□ 3□ 4 □ 5□ 6 □ 7□ 8 □ 9□ 10□
**I can hold my baby without assistance**	**After 2 hours**	0 □ 1□ 2□ 3□ 4 □ 5□ 6 □ 7□ 8 □ 9□ 10□
	**After 6 hours**	0 □ 1□ 2□ 3□ 4 □ 5□ 6 □ 7□ 8 □ 9□ 10□
	**After 24 hours**	0 □ 1□ 2□ 3□ 4 □ 5□ 6 □ 7□ 8 □ 9□ 10□
**I can feed/breastfeed my baby without assistance**	**After 2 hours**	0 □ 1□ 2□ 3□ 4 □ 5□ 6 □ 7□ 8 □ 9□ 10□
	**After 6 hours**	0 □ 1□ 2□ 3□ 4 □ 5□ 6 □ 7□ 8 □ 9□ 10□
	**After 24 hours**	0 □ 1□ 2□ 3□ 4 □ 5□ 6 □ 7□ 8 □ 9□ 10□
**I can take care of my personal hygiene/toilet**	**After 2 hours**	0 □ 1□ 2□ 3□ 4 □ 5□ 6 □ 7□ 8 □ 9□ 10□
	**After 6 hours**	0 □ 1□ 2□ 3□ 4 □ 5□ 6 □ 7□ 8 □ 9□ 10□
	**After 24 hours**	0 □ 1□ 2□ 3□ 4 □ 5□ 6 □ 7□ 8 □ 9□ 10□
**I feel in control**	**After 2 hours**	0 □ 1□ 2□ 3□ 4 □ 5□ 6 □ 7□ 8 □ 9□ 10□
	**After 6 hours**	0 □ 1□ 2□ 3□ 4 □ 5□ 6 □ 7□ 8 □ 9□ 10□
	**After 24 hours**	0 □ 1□ 2□ 3□ 4 □ 5□ 6 □ 7□ 8 □ 9□ 10□

Fourth tool: Maternal Functional Outcomes Assessment Tool: (developed by researchers)

Determine the degree of difficulty in performing daily activities after delivery:

Activities		(1) Inability to perform 0- > 12	(2) Partial dependence 13 - >24	(3) Full independence 25-36
Physical activity	Turn in bed			
	Get out of bed			
	Ambulation out of bed			
	Ability to get up from sitting position			
	Sitting in bed after surgery			
	Ability to return to sleeping position			
Self-care activity	Ability to urinate and defecate			
	Ability to shower and change clothes			
	Ability to eat food			
	Ability to take a nap			
Baby care	Ability to breastfeed the baby			
	Ability to change diapers			

The **total score ranges from 0 to 36**, with higher scores indicating better functional outcomes:

- **0–12**: Inability to perform daily activities, representing severe functional limitation.
- **13–24**: Partial dependence, indicating moderate functional impairment and the need for assistance.
- **25–36**: Full independence, reflecting optimal functional recovery and readiness to resume normal maternal roles

Nurse-Led Enhanced Recovery Program for Women undergoing Elective Cesarean Section: A quasi-experimental study.

Fifth Tool: Maternal Satisfaction with the ERP following Elective Cesarean Section

Adapted from: Morgan PJ, Halpern S, Lo J. The development of a maternal satisfaction scale for caesarean section. *Int J Obstet Anesth*. 1999;8(3):165-70. doi: 10.1016/s0959-289x(99)80132-0.

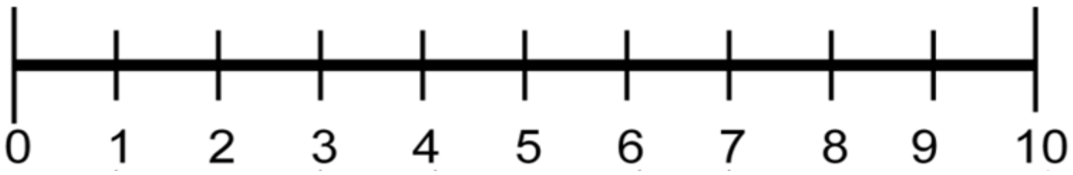

Not satisfied (0 - 4),

Neutral (5

Somewhat satisfied (6-9),

and "Completely satisfied (10).
